# Retraction: Prenatal Cocaine Exposure Upregulates BDNF-TrkB Signaling

**DOI:** 10.1371/journal.pone.0266626

**Published:** 2022-03-30

**Authors:** The PLOS ONE Editors

Following the publication of this article [1], concerns were raised regarding results presented in Figs 1, 2, 5, 6, 8, 9, 10, and 11.

Specifically:

1. The following panels appear similar:
  ○ The Fig 2A Hippocampus and Prefrontal Cortex β-actin panels and the Fig 2C Hippocampus and Prefrontal Cortex β-actin panels respectively.
  ○ The Fig 5A Hippocampus and Prefrontal Cortex TrkB panels and the Fig 6A Hippocampus and Prefrontal Cortex TrkB panels respectively.
  ○ The Fig 9A Hippocampus β-actin panel and the Fig 11A Hippocampus β-actin panel.
  ○ The Fig 10A Prefrontal cortex β-actin panel and the Fig 11B Prefrontal cortex β-actin panel.
2. When levels are adjusted to visualize background, the following panels appear to partially overlap:
  ○ The Fig 1B Hippocampus P75NTR panel and the Fig 1D Hippocampus ERK2 panel.
  ○ The Fig 1E Prefrontal cortex N-Shc panel and the Fig 1F Prefrontal cortex NR1 panel.
3. When levels are adjusted to visualize background, there appear to be vertical irregularities suggestive of splice lines in:
  ○ The Fig 5B Hippocampus pS^473^-Act1 panel, between lanes 2 and 3.
  ○ The Fig 6A Hippocampus NR1 panel, between lanes 2 and 3.
4. When levels are adjusted to visualize background, there appear to be horizontal and/or vertical irregularities in the background around bands presented in:
  ○ The Fig 8B Prefrontal cortex panel, between the two bands in lane 2.
  ○ The Fig 9A Hippocampus BDNF panel, between the BDNF bands in lanes 1–2, and around the BDNF bands in lanes 4–6.
  ○ The Fig 10A β-actin panel, around lane 3
  ○ The Fig 10A Prefrontal cortex Pro-BDNG panel, around the area presented below the 15kDa marker in lanes 2–6.
  ○ The Fig 11B Prefrontal cortex tPA panel, around the bands presented in lanes 5–6.

Regarding point 1, the corresponding author confirmed the duplications and explained that results in the indicated pairs of figure panels (Figs. 2A and 2C, Figs 9A and 11A, Figs 10A and 11B, and Figs 5A and 6A) originated from the same blots, which were re-probed with different antibodies.

The corresponding author disagreed with point 2 and stated that the results presented in these panels were obtained from separate blots.

Regarding points 3 and 4, the corresponding author indicated that the Fig 5B Hippocampus pS473-Act1 panel was spliced during figure preparation. The corresponding author disagreed with the concerns about irregularities in Figs 6A, 9A and 10A suggesting that the observations are likely the result of image artefacts or experimental artefacts such as gel or reagent remnants or protein degradation products.

The corresponding author provided image data to support their western blot results in this [1] and other *PLOS ONE* articles [2–5]. Per PLOS’ assessment of the data files, the pixel patterns in background areas of blot images provided for multiple panels in [1–5] appear more similar than would be expected for data obtained in independent experiments. The corresponding author stated that the repetitive features in the background noise of the underlying data are likely the result of scanner artifacts.

With the exception of point 1, the data and comments provided to PLOS did not resolve the concerns about the integrity and reliability of the reported data. Therefore, the *PLOS ONE* Editors retract this article.

HYW did not agree with the retraction and stands by the article’s findings. AS, KPB, and EF either did not respond directly or could not be reached.
